# Virtual follow‐up care among breast and prostate cancer patients during and beyond the COVID‐19 pandemic: Association with distress

**DOI:** 10.1002/cam4.6948

**Published:** 2024-03-11

**Authors:** Jacqueline L. Bender, Sarah Scruton, Geoff Wong, Nazek Abdelmutti, Alejandro Berlin, Julie Easley, Zhihui Amy Liu, Sharon McGee, Danielle Rodin, Jonathan Sussman, Robin Urquhart

**Affiliations:** ^1^ Cancer Rehabilitation and Survivorship, Department of Supportive Care Princess Margaret Cancer Centre Toronto Ontario Canada; ^2^ Dalla Lana School of Public Health University of Toronto Toronto Ontario Canada; ^3^ Institute of Health Policy, Management and Evaluation University of Toronto Toronto Ontario Canada; ^4^ Nuffield Department of Primary Care Health Sciences University of Oxford Oxford UK; ^5^ Cancer Quality Lab (CQual) Princess Margaret Cancer Centre Toronto Ontario Canada; ^6^ Cancer Digital Intelligence Princess Margaret Cancer Centre Toronto Ontario Canada; ^7^ Department of Radiation Oncology University of Toronto Toronto Ontario Canada; ^8^ Radiation Medicine Program Princess Margaret Cancer Centre Toronto Ontario Canada; ^9^ Department of Medical Education Horizon Health Network Fredericton New Brunswick Canada; ^10^ Biostatistics Department University Health Network Toronto Ontario Canada; ^11^ Division of Medical Oncology, Department of Medicine The Ottawa Hospital and the University of Ottawa Ottawa Ontario Canada; ^12^ Department of Oncology McMaster University Hamilton Ontario Canada; ^13^ Department of Community Health and Epidemiology Dalhousie University Halifax Nova Scotia Canada

**Keywords:** cancer, digital health, distress, follow‐up care, survivorship care, virtual care

## Abstract

**Background:**

The purpose of this study was to investigate associations between self‐reported distress (anxiety/depression) and satisfaction with and desire for virtual follow‐up (VFU) care among cancer patients during and beyond the COVID‐19 pandemic.

**Methods:**

Breast and prostate cancer patients receiving VFU at an urban cancer centre in Toronto, Canada completed an online survey on their sociodemographic, clinical, and technology, characteristics and experience with and views on VFU. EQ5D‐5 L was used to assess distress. Statistical models adjusted for age, gender, education, income and Internet confidence.

**Results:**

Of 352 participants, average age was 65 years, 48% were women,79% were within 5 years of treatment completion, 84% had college/university education and 74% were confident Internet users. Nearly, all (98%) had a virtual visit via phone and 22% had a virtual visit via video. The majority of patients (86%) were satisfied with VFU and 70% agreed that they would like VFU options after the COVID‐19 pandemic. Participants who reported distress and who were not confident using the Internet for health purposes were significantly less likely to be satisfied with VFU (OR = 0.4; 95% CI: 0.2–0.8 and OR = 0.19; 95% CI: 0.09–0.38, respectively) and were less likely to desire VFU option after the COVID‐19 pandemic (OR = 0.49; 95% CI: 0.30–0.82 and OR = 0.41; 95% CI: 0.23–0.70, respectively).

**Conclusions:**

The majority of respondents were satisfied with VFU and would like VFU options after the COVID‐19 pandemic. Future research should determine how to optimize VFU options for cancer patients who are distressed and who are less confident using virtual care technology.

## 1 INTRODUCTION

Breast and prostate cancer are the two most common cancers in men and women worldwide.^1^ Increasing 5‐year survival rates (e.g. 87% and 95% for breast and prostate cancer, respectively)^2^ have resulted in a growing number of cancer survivors with an estimated 158,430 breast and 176,360 prostate cancer survivors living in Canada today.^3^ This has increased the need for follow‐up care services after cancer treatment. Optimal follow‐up care involves (1) surveillance for cancer spread, recurrences or secondary cancers; (2) prevention and management of acute and persistent treatment side effects and (3) promotion of healthy behaviours to mitigate new and ongoing health concerns.^4^ In order for follow‐up care to be effective, cancer survivors require ongoing assessments and timely referrals to appropriate supportive care services to improve quality of life, reduce disability and restore function.^5^, ^6^

In Canada, follow‐up care is predominately delivered in‐person by oncologists in institution‐based settings. There is considerable evidence to demonstrate that this model of care is not working well for patients and is unsustainable.^7^ Specialist offices are often overcrowded and far from patients' homes and often have long wait times, short appointments and high costs per visit.^8^ In addition, many survivors report unmet practical, physical and psychological supportive care needs with this type of model.^9^, ^10^, ^11^ Alternatives, such as follow‐up with primary care providers or at primary care practices. Despite the fact that research has shown them to be effective in terms of patient satisfaction and disease control, many patients still say they prefer to be seen by a specialist for their follow‐up care.^12^, ^13^, ^14^ This may be due to beliefs about poorer quality and continuity of follow‐up care when it is with other care providers.^15^, ^16^

Virtual care, which is defined as remote interactions between patients and their providers using technology, has been promoted as a means to improve the experience and outcomes of follow‐up care by providing more accessible and patient‐centred care.^17^, ^18^ Prior to the COVID‐19 pandemic, use of software‐based virtual care was limited in Canada, as was the evidence on its effectiveness.^19^, ^20^, ^21^, ^22^ Since the COVID‐19 pandemic, however, there has been a major shift in Canada and elsewhere to providing care virtually by telephone, video‐conference, and online messaging, where possible.^23^ Emerging evidence on the outcomes of virtual care during the pandemic suggests that while the majority of patients have been satisfied with it, fewer would want virtual care again in the future.^24^, ^25^

Patient satisfaction is an important measure of healthcare quality as it assesses the extent to which a patient's needs and expectations were met.^26^ Limited research has examined the factors associated with patient satisfaction with and desire for virtual follow‐up (VFU), including the role of distress. Breast and prostate cancer patients commonly experience distress following treatment, and it is one of the most commonly reported symptoms by any type of cancer survivor.^27^, ^28^, ^29^ Experiencing distress is associated with poor quality of life and unmet needs following cancer treatment.^27^ Much of this distress comes from worry about the recurrence of cancer as well as long‐term side effects, which can be managed during follow‐up care.^29^, ^30^ To optimize VFU for cancer patients, we need to identify which patients and contexts would benefit from VFU. Thus, the purpose of this study was to investigate associations between self‐reported distress (anxiety/depression) and satisfaction with and desire for VFU during and beyond the COVID‐19 pandemic.

## 2 MATERIALS AND METHODS

### 2.1 Study design

We conducted a secondary analysis of a single‐centre, cross‐sectional virtual care evaluation survey of cancer patients who had received VFU (e.g., by phone and/or video conference) during the COVID‐19 pandemic. This study follows the STROBE reporting standards.^31^ This study was approved by the University Health Network (UHN) Quality Improvement Committee (QIRC) which granted this project a formal exemption from Research Ethics Board review (QI ID: 21‐00148). In the survey cover letter, participants were informed about the purpose of the study, what participation in the study involved, and how the data would be protected and used.

### 2.2 Setting and participants

The participants in this study included a sub‐sample of breast and prostate cancer patients who had completed the Virtual Care Evaluation Survey at the Princess Margaret Cancer Centre (PM) of the University Health Network in Toronto, Canada.^32^ The PM is a tertiary, university‐affiliated, teaching hospital, where virtual visits rose from 0.8% to 68.4% after the declaration of the COVID‐19 pandemic.^33^ To be eligible to participate in the survey, patients must have been diagnosed with cancer, above the age of 18, had received at least 1 appointment virtually (e.g. by phone and/or video) at the PM in the last 12 months and had a valid email address on file. Participants were eligible for the current sub‐study if they had been diagnosed with breast or prostate cancer, and were receiving post‐treatment follow‐up care at the time of the survey (i.e. were within 5 years of treatment completion).

### 2.3 Procedures

The PM Virtual Care Evaluation Survey was distributed to patients meeting the eligibility criteria between May and July 2021. Patients received an email invitation to complete the anonymous online survey with a link to the secure, web‐based REDCap platform hosted at the University Health Network. Non‐responders were sent two follow‐up email invitations.

### 2.4 Measures

#### 2.4.1 Virtual care

Patients were asked to indicate the type of virtual care appointments they had received in the past 12 months (e.g. phone, video), their overall satisfaction with the virtual care they had received, their satisfaction with phone vs video‐based virtual care specifically, and their desire for virtual care after the COVID‐19 pandemic. The two main outcomes of this study were overall satisfaction with virtual care and desire for virtual care following the COVID‐19 pandemic. The former was measured by asking “Overall, how satisfied are you with the virtual care you received at Princess Margaret?” which included a 5‐point Likert response option (Very satisfied, satisfied, neutral, dissatisfied, very dissatisfied). The latter was measured by asking respondents to rate their level of agreement with the statement “I would like to continue to have virtual options for some of my visits after the COVID‐19 pandemic ends”. This question also included a 5‐point Likert response option (strongly agree, agree, neutral, disagree, strongly disagree). For this analysis, both responses were transformed into binary variables (e.g. satisfied/very satisfied vs. neutral/dissatisfied/very dissatisfied).

#### 2.4.2 Distress

Distress was measured with the “Anxiety/Depression” sub‐scale of the EuroQoL (EQ5D‐5L)—a widely used 5‐item measure of health‐related quality‐of‐life (1, no problems, to 5, severe problems).^34^ Response options included: I am not anxious or depressed, I am slightly anxious or depressed, I am moderately anxious or depressed, I am severely anxious or depressed, I am extremely anxious or depressed. For the univariable and multivariable analyses, responses were collapsed into a binary variable: no anxiety/depression versus any level of anxiety/depression (from slightly to extreme).

#### 2.4.3 Sociodemographic, clinical and technology characteristics

Sociodemographic characteristics included age (continuous), gender identity (man, woman, non‐binary), whether they were born in Canada (yes/no), whether they spoke English as a first language (yes/no), race/ethnicity (10 response options), highest level of education (high school or less, college/technical school, university undergraduate, postgraduate) and household income (less than $60,000, $60,000–$100,000, more than $100,000). Health literacy was measured with the Single Item Literacy screener to capture functional health literacy: How often do you need assistance when reading instructions or written material for your doctor or pharmacy?^35^ Response options were: never, rarely, sometimes, often, always. Clinical characteristics included phase in the cancer journey (less than 3 months after treatment, between 3 months and less than 5 years after treatment and more than 5 years after treatment) and type of treatment (surgery, radiation therapy, chemotherapy, hormone therapy). Technology access and literacy were assessed with two questions: do you have access to a phone, tablet or computer with Internet access (yes/no), and how confident are you using information from the Internet for health purposes? (not confident, somewhat confident, neutral, confident, very confident). The latter was adapted from the final item of the eHealth literacy scale.^36^

### 2.5 Data analysis

Participant characteristics and study outcomes (satisfaction with VFU and desire for VFU after COVID‐19) were summarized using descriptive statistics for the whole sample and stratified by cancer type. Univariable logistic regression was used to assess associations between participant sociodemographic, clinical and technology characteristics identified a priori and each of the two outcomes of interest. Multivariable logistic regression models were constructed to assess variables that were significantly associated with our outcomes of interest while adjusting for age, education and income, which are known determinants of technology use, as well as gender given that coping styles can be influenced by gender norms.^37^, ^38^ A *p* value of <0.05 is considered as statistically significant. All analyses were conducted in R version 4.1.2.^39^

## 3 RESULTS

Of the 2343 participants who had completed the PM Virtual Care Survey, there were 633 with breast or prostate cancer. An additional 281 individuals were excluded as they had not finished treatment within the past 5 years, or they did not receive any VFU appointments during the study period, leaving a final sample of 352 participants. The mean age of participants was 64.5 years. Prostate cancer patients made up 51% of the sample and breast cancer patients made up 49%, 1% of which identified as a man. The cohort was 79% white, and the majority had high education, household income and health literacy. Just over half (55%) of participants indicated that they did not experience any anxiety or depression. Detailed participant demographics can be found in Table 1.

**TABLE 1:** Frequency and prevalence of patient sociodemographic and clinical characteristics.

Characteristics	Count (%) (unless otherwise specified)		
	Full population	Breast cancer	Prostate cancer
	*n* = 352	*n* = 172	*n* = 180
Sociodemographic characteristics			
Age, mean (SD)	65.4 (10.7)	60.3 (11.1)	70.2 (7.6)
Gender identity			
Man	180 (52)	2 (1)	178 (100)
Woman	169 (48)	169 (99)	0 (0)
Born in Canada			
Yes	214 (61)	110 (64)	104 (58)
No	137 (39)	61 (36)	76 (42)
English as a first language			
Yes	261 (75)	124 (73)	137 (77)
No	89 (25)	47 (27)	42 (23)
Race/Ethnicity			
White/Caucasian/European	278 (79)	130 (76)	148 (82)
East Asian	25 (7)	17 (10)	8 (4)
Black/African	16 (5)	9 (5)	7 (4)
Other	33 (9)	16 (9)	17 (9)
Highest level of education			
University	137 (39)	65 (38)	72 (40)
Postgraduate	98 (28)	47 (27)	51 (29)
College	61 (17)	35 (20)	26 (15)
High school or less	53 (15)	24 (14)	29 (16)
Household income			
Less than $60,000	62 (18)	33 (19)	29 (16)
$60,000–100,000	68 (19)	34 (20)	34 (19)
$100,000 or more	150 (43)	71 (41)	79 (44)
Prefer not to say	72 (20)	34 (20)	38 (21)
Need of assistance when reading instructions/written material from doctor or pharmacy			
Often/Always	16 (5)	5 (3)	11 (6)
Sometimes/Rarely/Never	336 (95)	167 (97)	169 (94)
Clinical characteristics			
Phase in cancer journey			
Less than 5 years after treatment	223 (63)	118 (69)	105 (58)
More than 5 years after treatment	72 (20)	20 (12)	52 (29)
Less than 3 months after treatment	57 (16)	34 (20)	23 (13)
Type of treatment			
Chemotherapy	93 (26)	86 (50)	7 (4)
Hormone therapy	125 (36)	71 (41)	54 (30)
Radiation therapy	224 (69)	140 (81)	104 (58)
Surgery	227 (64)	143 (83)	84 (47)
Anxiety/Depression			
I am not anxious or depressed	194 (55)	78 (46)	116 (64)
I am slightly anxious or depressed	109 (31)	58 (34)	51 (28)
I am moderately anxious or depressed	40 (11)	28 (16)	12 (7)
I am severely anxious or depressed	6 (2)	5 (3)	1 (1)
I am extremely anxious or depressed	2 (1)	2 (1)	0 (0)
Technology characteristics			
Access to a phone tablet or computer with internet at home			
Yes	345 (99)	170 (99)	176 (98)
No	5 (1)	1 (1)	4 (2)
Confidence using the internet for health‐related purposes			
Confident/very confident	259 (74)	122 (71)	137 (76)
Neutral/somewhat confident/not confident	91 (26)	49 (29)	42 (24)

### 3.1 VFU use

Within the last year, nearly all participants (98%) had at least one virtual visit via phone and 22% had at least one virtual visit via video. Of the prostate cancer patients, 100% had a virtual visit by phone, whereas only 4% had a video visit. In contrast, 97% of breast cancer patients had a phone visit and 41% had a video visit. Of those who received phone visits, only 16% and 8% of breast and prostate cancer patients, respectively, were not fully satisfied. Of those who received video visits, 21% of breast cancer patients were not fully satisfied. There were no prostate cancer patients who indicated that they were not satisfied with video visits, but this may be because very few prostate patients received them (*n* = 8). Detailed descriptive statistics for VFU use can be found in Table 2.

**TABLE 2:** Virtual care characteristics.

Characteristics	Count (%) (unless otherwise specified)		
	Full population	Breast cancer	Prostate cancer
	*n* = 352	*n* = 172	*n* = 180
Type of appointment received in the past 12 months			
Phone	346 (98)	166 (97)	180 (100)
Video	78 (22)	70 (41)	8 (4)
Overall satisfaction with virtual care			
Satisfied/very satisfied	296 (86)	139 (82)	157 (89)
Neutral/dissatisfied/very dissatisfied	50 (14)	30 (18)	20 (11)
Overall satisfaction with phone visits			
Satisfied/very satisfied	302 (88)	138 (84)	164 (92)
Neutral/dissatisfied/very dissatisfied	42 (12)	27 (16)	15 (8)
Overall satisfaction with video visits			
Satisfied/very satisfied	62 (81)	55 (79)	7 (100)
Neutral/dissatisfied/very dissatisfied	15 (19)	15 (21)	0 (0)
Agreement with the following statement: I would like to have virtual options for some of my visits COVID‐19			
Agree/strongly agree	247 (70)	113 (66)	134 (70)
Neutral/disagree/strongly disagree	103 (30)	59 (34)	45 (25)

### 3.2 Satisfaction with VFU

Eighty‐six per cent (82% of breast cancer patients and 89% of prostate cancer patients) were satisfied/very satisfied with virtual care overall. In the univariable analysis, satisfaction with VFU was associated with having anxiety or depression and confidence using the internet. Those who indicated that they experienced anxiety or depression had a lower odds of being satisfied with VFU compared to those who did not have anxiety or depression (OR = 0.37; 95% CI: 0.19–0.68). In addition, those who felt neutral, somewhat confident, or not confident using the internet for health‐related purposes had a lower odds of being satisfied with VFU compared to those who felt confident or very confident (OR = 0.21; 95% CI: 0.11–0.38). The results of the multivariable analysis were very similar; having anxiety/depression and low confidence using the internet for health‐related purposes were the only two factors significantly associated with lower satisfaction with VFU (OR = 0.4; 95% CI: 0.2–0.8 and OR = 0.19; 95% CI: 0.09–0.38, respectively). The results of these models are displayed in Table 3.

**TABLE 3:** Univariate (unadjusted) and multivariable (adjusted) logistic regression analyses.

Covariate comparisons	Satisfaction with virtual care				Desire for virtual care after COVID‐19			
	Unadjusted		Adjusted		Unadjusted		Adjusted	
	OR (95% CI)	*p* Value	aOR (95% CI)	*p* Value	OR (95% CI)	*p* Value	aOR (95% CI)	*p* Value
Age in years	1.02 (0.99,1.05)	0.19	1.03 (0.99, 1.07)	0.15	1.00 (0.97, 1.02)	0.69	0.99 (0.97, 1.02)	0.58
Gender (compared to male)								
Female	0.59 (0.32, 1.08)	0.086	1.08 (0.49, 2.35)	0.85	0.66 (0.42, 1.05)	0.08	0.70 (0.40, 1.25)	0.23
First language (compared to English)								
Other language	0.70 (0.37, 1.35)	0.29	‐	‐	0.88 (0.52, 1.49)	0.64	‐	‐
Ethnicity (compared to White/Caucasian/European)								
East Asian	0.78 (0.25, 2.41)	0.67	‐	‐	0.62 (0.26, 1.47)	0.27	‐	‐
Black/African	0.62 (0.17, 2.32)	0.48	‐	‐	0.62 (0.22, 1.75)	0.36	‐	‐
Other	0.70 (0.27, 1.82)	0.47	‐	‐	0.50 (0.24, 1.05)	0.067	‐	‐
Education (compared to high school or less)								
College	0.84 (0.33, 2.12)	0.72	0.74 (0.25, 2.15)	0.58	1.57 (0.72, 3.41)	0.26	1.63 (0.70, 3.84)	0.26
University	1.79 (0.75, 4.24)	0.19	1.53 (0.56, 4.20)	0.41	1.65 (0.85, 3.20)	0.14	1.28 (0.61, 2.69)	0.51
Postgraduate school	1.84 (0.72, 4.68)	0.20	1.46 (0.49, 4.36)	0.50	1.89 (0.93, 3.86)	0.081	1.51 (0.67, 3.36)	0.32
Household income (compared to less than $60,000)								
$60,000–100,000	2.06 (0.75, 5.62)	0.16	1.66 (0.55, 5.02)	0.37	1.01 (0.50, 2.07)	0.97	0.72 (0.33, 1.58)	0.41
$100,000 or more	1.44 (0.66, 3.15)	0.36	0.93 (0.37, 2.33)	0.88	2.16 (1.13, 4.12)	0.02	1.59 (0.77, 3.25)	0.21
I prefer not to say	1.44 (0.57, 3.61)	0.44	1.75 (0.60, 5.10)	0.30	1.18 (0.58, 2.40)	0.65	1.10 (0.51, 2.38)	0.8
Health literacy (compared to low health literacy)								
High health literacy	2.88 (0.96, 8.67)	0.06	‐	‐	1.45 (0.51, 4.10)	0.48	‐	‐
Phase of cancer journey (compared to <5 years after treatment)								
>5 Years after treatment	0.76 (0.36, 1.58)	0.46	‐	‐	0.93 (0.52, 1.67)	0.82	‐	‐
<3 Months after treatment	0.76 (0.34, 1.72)	0.51	‐	‐	0.94 (0.50, 1.79)	0.86	‐	‐
Receipt of chemotherapy (compared to no)								
Yes	0.67 (0.35, 1.27)	0.22	‐	‐	0.74 (0.44, 1.23)	0.24	‐	‐
Receipt of hormone therapy (compared to no)								
Yes	0.55 (0.30, 1.01)	0.055	‐	‐	0.79 (0.49, 1.27)	0.33	‐	‐
Receipt of radiation therapy (compared to no)								
Yes	0.76 (0.38, 1.49)	0.43	‐	‐	0.63 (0.37, 1.05)	0.078	‐	‐
Receipt of surgery (compared to no)								
Yes	0.92 (0.49, 1.74)	0.80	‐	‐	1.08 (0.67, 1.74)	0.76	‐	‐
Anxiety/depression (compared to not having anxiety/depression)								
Having anxiety/depression	0.37 (0.19, 0.68)	0.002	0.40 (0.20, 0.80)	0.010	0.45 (0.28, 0.72)	<0.001	0.49 (0.30, 0.82)	0.006
Confidence using the Internet for health‐related purposes (compared to confident/very confident)								
Neutral/somewhat confident/not confident	0.21 (0.11, 0.38)	<0.001	0.19 (0.09, 0.38)	<0.001	0.39 (0.23, 0.64)	<0.001	0.41 (0.23, 0.70)	0.0014

### 3.3 Desire for VFU after COVID‐19

Seventy per cent (66% of breast cancer patients and 70% of prostate cancer patients) agreed or strongly agreed that they would want VFU appointments to continue after COVID‐19. In the univariable analysis, less desire for VFU after COVID‐19 was associated with the same factors as satisfaction with VFU: having anxiety/depression and being neutral, somewhat confident or not confident using the internet for health‐related purposes (OR = 0.45; 95% CI: 0.28–0.72 and OR = 0.39; 95% CI: 0.23–0.64, respectively). A multivariable analysis was performed using the same variables that were included in the multivariable analysis of satisfaction with VFU, and the results were the same; having anxiety/depression and low confidence using the internet for health‐related purposes were the only two factors significantly associated with higher satisfaction with desire for VFU after the COVID‐19 pandemic (OR = 0.49; 95% CI: 0.30–0.82, and OR = 0.41; 95% CI: 0.23–0.70, respectively). The results of these models are displayed in Table 3.

## 4 DISCUSSION

During the COVID‐19 pandemic virtual care went from being rare in Canada to being a necessity. This experience has demonstrated the possibilities for system‐wide change and the opportunity to sustain virtual care options.^40^ This study has demonstrated that there is considerable patient interest in sustaining VFU options. However, breast and prostate cancer patients who were distressed and those less confident using the Internet were less likely to be satisfied with VFU during the COVID‐19 pandemic and less likely to desire VFU options after the COVID‐19 pandemic. Cancer‐related distress is highly prevalent across the cancer continuum and strategies for early and ongoing intervention are critical.^29^ These findings highlight the need to optimize the delivery of VFU for patients who are distressed or provide alternative options for care.

Although the majority (86%) of breast and prostate cancer patients were satisfied with VFU, 16% fewer would want VFU in the future. While these findings align with trends in Canada and elsewhere, the proportion of cancer patients who reported that they would be interested in VFU beyond COVID‐19 is higher than that reported in other studies.^33^, ^41^, ^42^ For example, of 343 breast cancer survivors receiving telephone‐VFU during the COVID‐19 pandemic in Italy, 80.3% were satisfied, but only 43.8% would want it in the future.^24^ Likewise, in study of 155 cancer patients receiving virtual cancer rehabilitation during the COVID‐19 pandemic in the U.S., 94.8% agreed that it was good experience, but only 63.1% reported that they would be interested in using it the future.^43^ These findings suggest that virtual care during a pandemic is acceptable to cancer patients but may be a less ideal form of care when there are no safety concerns associated with in‐person appointments.

Where patients are in the cancer journey influences their preferences for virtual care. In a study of 397 cancer patients receiving virtual care during the COVID‐19 pandemic in Alberta Canada, patients receiving follow‐up care were more satisfied with virtual care than those on treatment (83.6% vs. 73.2%, *p* < 0.05).^41^ Similarly, of 2343 patients and 100 physicians participating in the parent PM Virtual Care study, patients who recently completed treatment were two times more likely to prefer in‐person visits compared to those in long‐term (5 or more years) follow‐up.^32^ Additionally, long‐term follow‐up care was selected by both patients and physicians as the most appropriate type of visit for virtual visits.^32^ Given the urgent need to find alternatives to in‐person specialist follow‐up care, these findings are promising and suggest that virtual care may be more acceptable to patients in the follow‐up care phase of the cancer trajectory.

Breast and prostate cancer patients who reported distress (which corresponded to 45% of the sample) were about 63% less likely to be satisfied with VFU and 55% less likely to want VFU in the future. Two other studies have reported an association between cancer patient distress and satisfaction with or preferences for VFU. In a multi‐centre study of virtual care during the COVID‐19 pandemic involving 1299 breast cancer survivors from 18 hospitals in France and Italy, having minimal to severe anxiety (as measured by HADS) was associated with significantly lower levels of virtual care satisfaction.^44^ Likewise, in a study of 1382 cancer patients in British Columbia Canada, having low mental health scores (as measured by the VR‐12 MCS sub‐scale) was associated with lower preferences for virtual visits if offered after the COVID‐19 pandemic.^42^ The association between increased distress and less satisfaction with VFU may be due to the perception that physicians provide better emotional support during in‐person visits, which is important to those experiencing anxiety or depression. A subsequent qualitative investigation involving a sub‐group of survey respondents found that patients feel more comforted during in‐person visits because they have more time to discuss personal or emotional topics with their physician compared to virtual visits, as well as the ability to read visual cues and body language.^45^ In addition, patients may rely on physical exams during in‐person appointments to lessen their anxiety and fear of recurrence.

Screening for distress is widely recognized as a standard component of quality cancer care, along with referral to relevant supportive care programming.^46^ As those who experienced distress were less likely to be satisfied with VFU and therefore may not be a good candidate for this type of care, it is important to ensure individuals are properly screened in advance. Problematically, screening for distress was disrupted with the transition to virtual visits during the COVID‐19 pandemic, as many centres depend on in‐person workflows to implement patient‐reported outcomes. For example, a review of administrative data in the province of Alberta in Canada revealed that distress screening rates dropped from 70% pre‐pandemic to 15% during the pandemic, and supportive care referral rates dropped from a ratio of 5 to 1.^47^ Screening for distress may be even more important in virtual visits as clinicians have fewer non‐verbal cues at their disposal to help them assess the wellbeing of the patient.^47^ This may in part explain why only 31.5% of 396 Alberta healthcare providers surveyed reported feeling confident meeting patients' emotional needs virtually, and why patients report dissatisfaction with the lack of emotional support provided during virtual visits.^41^

Previous studies of cancer patients' experiences with virtual care during the COVID‐19 pandemic indicate that it may disproportionately benefit more advantaged patients and widen the digital divide. In this study, cancer patients who were less confident using the Internet for health‐related purposes, were less likely to be satisfied with VFU during the pandemic and to desire VFU after the pandemic, however, no other sociodemographic variables were significant. In their study of cancer patients in British Columbia, Izadi‐Najafabadi et al. found that lower perceived ease of use of telehealth and less education were associated with cancer patients' satisfaction with virtual care across the continuum of care.^42^ Additionally, older age, female sex, non‐white race, lower education, and living in an urban environment were associated with a lower likelihood of wanting telehealth after the pandemic. An earlier study by Berlin et al. of patients at various phases of the cancer journey recruited from the same institution found that patient satisfaction with virtual care was associated with sex and income, but not with age or equity indexes.^33^ However, ethnocultural composition (self‐identification as a visible minority, foreign‐born, linguistic isolation and recent immigration) was associated with a lower likelihood of requesting future virtual care in the future. In addition, a study of individuals with hematological malignancies reported that the use of virtual care was associated with higher residential stability (e.g. owning versus renting a home).^48^

Optimization of virtual care infrastructure, provision of virtual care technology options, and technical support are needed to ensure that all patients have access to high‐quality VFU if desired. This includes the continued use of the more familiar and accessible telephone follow‐up visit, which was used by 98% of the study sample, in comparison to video visits which were used by only 22%. However, changes to the virtual care funding model in Ontario (where this study took place) aim to limit telephone‐based care by reducing the amount physicians get paid for telephone visits to 85% of the fee they can bill for an in‐person visit for patients with an existing relationship with a physician, while video visits can be billed at the same amount.^49^ This will likely cause some providers to limit their use of telephone‐based follow‐up. These changes will make it difficult for people without high‐speed Internet and who cannot afford the technology required for video visits to access virtual care. While universal broadband, is a strategic priority in Canada,^50^ only 59.5% of rural and remote communities and 42.9% of indigenous communities have access to high‐speed Internet^51^ which would be required for a video visit.

### 4.1 Study limitations

This study has some important limitations. We recruited from a single urban North American cancer centre, limiting generalizability. In addition, the study sample has predominantly consisted of white, English‐speaking, and/or highly educated individuals, which may introduce a potential bias and limit the applicability of our results to other populations. The survey was distributed online and patients had to have an email address to participate, which may have introduced selection bias towards cancer patients who are more confident in using the Internet. The abbreviated EQ5D‐5L measure was used to assess distress, instead of a more robust multi‐dimensional measure; though the findings align with previous studies using other measures of distress. As the survey was cross‐sectional, information on baseline distress was not available and timing of distress with receipt of VFU was not possible.

### 4.2 Conclusion

The forced transition to virtual care during the COVID‐19 pandemic provided an excellent opportunity to assess the feasibility and acceptability of VFU among cancer patients. The high proportion of breast and prostate cancer patients in this study who were interested in VFU after the pandemic, provides evidence in favour of sustaining VFU options post‐pandemic to address the challenges with the current model of in‐person follow‐up care delivered by oncology specialists. Future efforts should determine how to optimize VFU for cancer patients who are distressed and who are less confident using virtual care technology and ensure virtual care funding policies enable all patients to have access to low and high‐tech VFU when needed.

## AUTHOR CONTRIBUTIONS

**Jacqueline L. Bender:** Conceptualization (lead); data curation (equal); formal analysis (lead); funding acquisition (lead); investigation (lead); methodology (lead); project administration (lead); resources (lead); software (lead); supervision (lead); validation (lead); writing – original draft (lead); writing – review and editing (lead). **Sarah Scruton:** Data curation (equal); formal analysis (equal); investigation (equal); methodology (equal); project administration (equal); validation (equal); writing – original draft (equal); writing – review and editing (equal). **Geoff Wong:** Conceptualization (equal); formal analysis (equal); investigation (equal); methodology (equal); validation (equal); writing – original draft (supporting); writing – review and editing (equal). **Nazek Abdelmutti:** Conceptualization (equal); data curation (equal); formal analysis (equal); investigation (equal); methodology (equal); validation (equal); writing – original draft (supporting); writing – review and editing (equal). **Alejandro Berlin:** Conceptualization (equal); data curation (equal); formal analysis (equal); investigation (equal); methodology (equal); validation (equal); writing – original draft (supporting); writing – review and editing (equal). **Julie Easley:** Conceptualization (equal); formal analysis (equal); investigation (equal); methodology (equal); validation (equal); writing – original draft (supporting); writing – review and editing (equal). **Zhihui Amy Liu:** Conceptualization (equal); data curation (equal); formal analysis (equal); investigation (equal); methodology (equal); validation (equal); writing – original draft (supporting); writing – review and editing (equal). **Sharon McGee:** Conceptualization (equal); formal analysis (equal); investigation (equal); methodology (equal); validation (equal); writing – original draft (supporting); writing – review and editing (equal). **Danielle Rodin:** Conceptualization (equal); formal analysis (equal); investigation (equal); methodology (equal); validation (equal); writing – original draft (supporting); writing – review and editing (equal). **Jonathan Sussman:** Conceptualization (equal); formal analysis (equal); investigation (equal); methodology (equal); validation (equal); writing – original draft (supporting); writing – review and editing (equal). **Robin Urquhart:** Conceptualization (equal); formal analysis (equal); investigation (equal); methodology (equal); validation (equal); writing – original draft (supporting); writing – review and editing (equal).

## FUNDING INFORMATION

This study was funded by the Canadian Institutes of Health Research (grant #TT7 128272).

## CONFLICT OF INTEREST STATEMENT

The authors declare no conflict of interest. The funder had no role in the design, execution, interpretation or writing of the study.

## Data Availability

The data that support the findings are available upon request from the corresponding author.
